# Computation of expected values of some connectivity based topological descriptors of random cyclooctane chains

**DOI:** 10.1038/s41598-024-57175-y

**Published:** 2024-04-02

**Authors:** Shamaila Yousaf, Zaffar Iqbal, Saira Tariq, Adnan Aslam, Fairouz Tchier, Abudulai Issa

**Affiliations:** 1 Department of Mathematics, University of Gujrat, Hafiz Hayat Campus, Gujrat, Pakistan; 2 Department of Natural Sciences and Humanities, University of Engineering and Technology, Lahore (RCET), Pakistan; 3 Henan International Joint Laboratory for Multidimensional Topology and Carcinogenic Characteristics Analysis of Atmospheric Particulate Matter PM2.5, Pingdingshan, 467000 China; 4 Mathematics Department, College of Science, King Saud University, P.O. Box 22452, 11495 Riyadh, Saudi Arabia; 5 Department of Mathematics, University of Lome, P. O. Box 1515, Lome, Togo

**Keywords:** Chemical graph theory, Topological indices, Cyclooctane chains, Analytical chemistry, Theoretical chemistry

## Abstract

Cyclooctane is a cycloalkane consisting of carbon and hydrogen atoms arranged in a closed ring structure. Cyclooctane chains can be found in various organic compounds and are significant in the field of organic chemistry due to their diverse reactivity and properties. The atom-bond connectivity index ($\mathcal{A}\mathcal{B}\mathcal{C}$), the geometric-arithmetic index ($\mathcal{G}\mathcal{A}$), the arithmetic–geometric index ($\mathcal{A}\mathcal{G}$) and the forgotten index ($\mathcal{F}$) are four well-studied molecular descriptors that have found applications in QSPR and QSAR studies. These topological descriptors have shown significant correlations with different physiochemical properties of octane isomers. In this work, the expected values of four degree based topological descriptors for random cyclooctane chains are calculated. An analytical comparison is given between the expected values of $\mathcal{A}\mathcal{B}\mathcal{C}$, $\mathcal{G}\mathcal{A}$, $\mathcal{A}\mathcal{G}$, and $\mathcal{F}$ indices of random cyclooctane chains.

## Introduction

A significant branch of mathematics that deals with mathematical models of graphs is called chemical graph theory. It is a branch of mathematics that combines chemistry and graph theory. The physical and chemical properties of molecules correspond with their molecular geometry, which is derived from the vast amount of data used for the analysis. Generally, the characteristics of a molecule obtained through chemical examinations can be efficiently determined by calculating the topological indices. In many cases, theoretical chemistry plays a vital role in chemical graph theory.

Molecular graph theory is a branch of theoretical chemistry that represents chemical compounds, specifically molecules, as graphs. In this context, a graph is a mathematical structure composed of vertices (atoms) and edges (bonds) that connect these vertices. The representation allows for the abstraction and analysis of molecular structures, facilitating the study of various properties and behaviours of chemical compounds. Let $\Pi =\Pi (V, E)$ be a simple, finite connected graph of order n with vertex set $V\left(\Pi \right)$ and an edge set $E\left(\Pi \right)$. The degree of a vertex ${u}_{i}$ is denoted by $d_{i}$ and is defined as the number of edges incident to it. For undefined notions related to graph theory see^1^.

Molecular descriptors are the numerical or categorical representations of the structural and chemical features of a molecule. These molecular descriptors are important in the field of computational chemistry and biology. They provide information about molecular size, chemical composition, shape and other relevant properties of the molecular structure which may be used for designing drug, predicting toxicity and quantitative structure property relationship studies. Numerous molecular descriptors have been introduced by different researchers and are important in studying the characteristics of chemical structures. The first distance based topological index was introduces by H. Wiener^2^, while he was working on the boiling point of paraffin. The Zagreb indices and their variants^3^ are among the most studied degree based topological indices. They have been used to study branching problem in the early seventeen century. Consider the following general graph invariant

$$I\left(\Pi \right)=\sum_{{u}_{i}{u}_{j}\epsilon E\left(\Pi \right)}f({d}_{i},{d}_{j}).$$

A well-known topological index called the $\mathcal{F}$ index was introduced by Furtula and Gutman^4^ in 2015. It is defined as the sum of squares of degrees of the vertices of chemical graphs.

1 $$\mathcal{F}\left(\Pi \right)=\sum_{{u}_{i},{u}_{j}\epsilon E\left(\Pi \right)}{{d}_{i}}^{2}+{{d}_{j}}^{2}$$

It was observed that the predictive ability of $\mathcal{F}$ index and the first Zagreb index is same. The correlation coefficient of $\mathcal{F}$ index for the properties acentric factor and entropy is greater than 0.95.

The Atom-Bond Connectivity index^5^ was proposed in 1998 by a Cuban mathematician named Ernesto Estrada. The $\mathcal{A}\mathcal{B}\mathcal{C}$ index is a helpful predictive index that is used to study the formation of heat in alkanes. It is defined as.

2 $$\mathcal{A}\mathcal{B}\mathcal{C}\left(\Pi \right)=\sum_{{u}_{i},{u}_{j}\epsilon E\left(\Pi \right)}\sqrt{\frac{{d}_{i}+{d}_{j}-2}{{d}_{i}{d}_{j}}}.$$

Vukičević and Furtula^6^ introduced a topological index called the geometric-arithmetic index $\mathcal{G}\mathcal{A}$. The geometric-arithmetic index is a very useful tool in the investigations of QSAR and QSPR studies. It is defined as

3 $$\mathcal{G}\mathcal{A}\left(\Pi \right)=\sum_{{u}_{i},{u}_{j}\epsilon E\left(\Pi \right)}\frac{2\sqrt{{d}_{i}{d}_{j}}}{{d}_{i}+{d}_{j}}.$$

The arithmetic–geometric index $\mathcal{A}\mathcal{G}$^7^ was recently introduced as a modification of the well-known geometric–arithmetic index $\mathcal{G}\mathcal{A}$. This is defined by

4 $$\mathcal{A}\mathcal{G}\left(\Pi \right)=\sum_{{u}_{i},{u}_{j}\epsilon E\left(\Pi \right)}\frac{{d}_{i}+{d}_{j}}{2\sqrt{{d}_{i}{d}_{j}}}.$$

For more details on the computations of topological indices for different chemical structures, see^8–11^.

In this work, we compute the expected values of four degree based topological indices for the class of random cyclooctane chain: the atom-bond connectivity index, the arithmetic–geometric index, the geometric- arithmetic index, and the forgotten index. An analytical comparison between the expected value of these topological indices with same probability has been given. More precisely, we have proved that the expected value of $\mathcal{A}\mathcal{B}\mathcal{C}$ index is always less than the expected value of $\mathcal{G}\mathcal{A}$ index and that the expected value of $\mathcal{A}\mathcal{G}$ index is less than the expected value of forgotten index.

## Random cyclooctane chain

A cyclooctane is a cycloalkane which is a type of saturated hydrocarbon with eight carbon atoms arranged in a cycle. It has a chemical formula of C_8_H_16_. Cyclooctane is a stable and nonpolar compound with a simple structure. In organic chemistry, it is used as a reference compound and is part of different organic molecules and reactions. The simple and symmetrical ring structure of cyclooctanes make them ideal model to understand the properties of cyclic hydrocarbons. The study of cyclooctane and its derivatives is important in stereochemistry, particularly when it comes to puckering conformational changes in cycloalkanes. The simple structure and reactivity of cycloocatne make it an important reference point for researchers.

For a long time, chemists gave more attention to the derivatives of saturated hydrocarbons, which are used in drug synthesis, kinetic combustion, and organic synthesis etc. For example, they are used as reagents, synthetic organics. They are also used in the production of adhesives, coatings, and many other purposes. Some scientists got interested in octagonal graphs^12^. Brunvoll et al.^1^ studied the number of isomers in octagonal graphs. Many scientists showed their interest in the topological indices of cyclooctanes. Shouliu Wei et. al.^13^ calculated the Wiener indices of cyclooctanes. Three types of Kirchoff indices of cyclooctanes have been determined by Yoy Linhua et. al. in^14^. Jia-Bao Liu et. al.^15^ calculated the Gutman index and Schultz index of the cyclooctane chains. Recently, Zahid Raza et. al.^16^ calculated some topological index such as harmonic index and sum-connectivity index of cyclooctane chains. Liu H. et. al. have computed some expected values of sombor indices of hexagonal chains, phenylene graphs^17^. For more details, see^8–11,18–30^.

## Materials and methods

**Figure 1:**
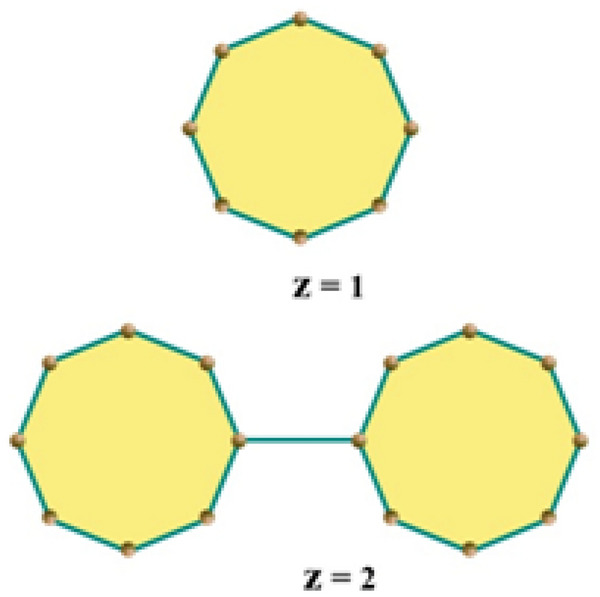
The cyclooctane chains for $z = 1,2.$

**Figure 2:**
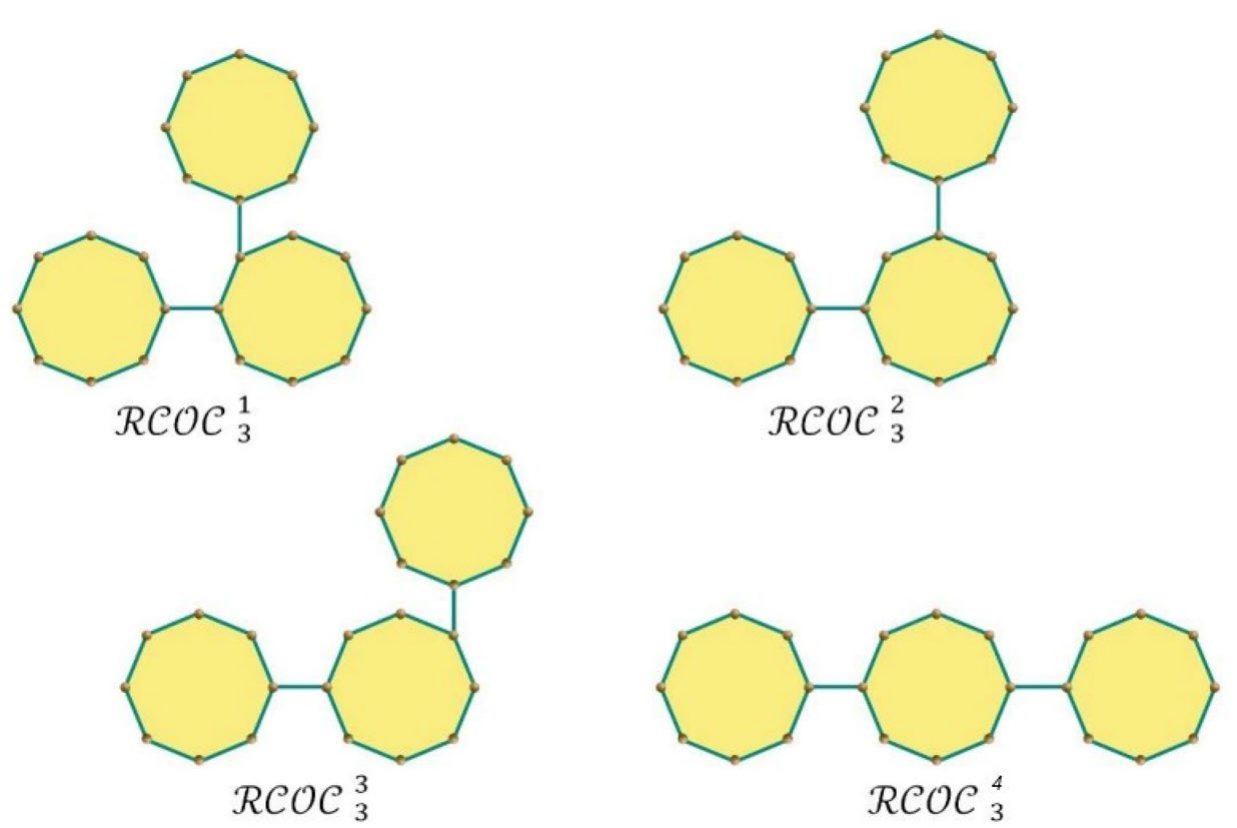
The four type of cyclooctane chains for $z = 3.$

**Figure 3:**
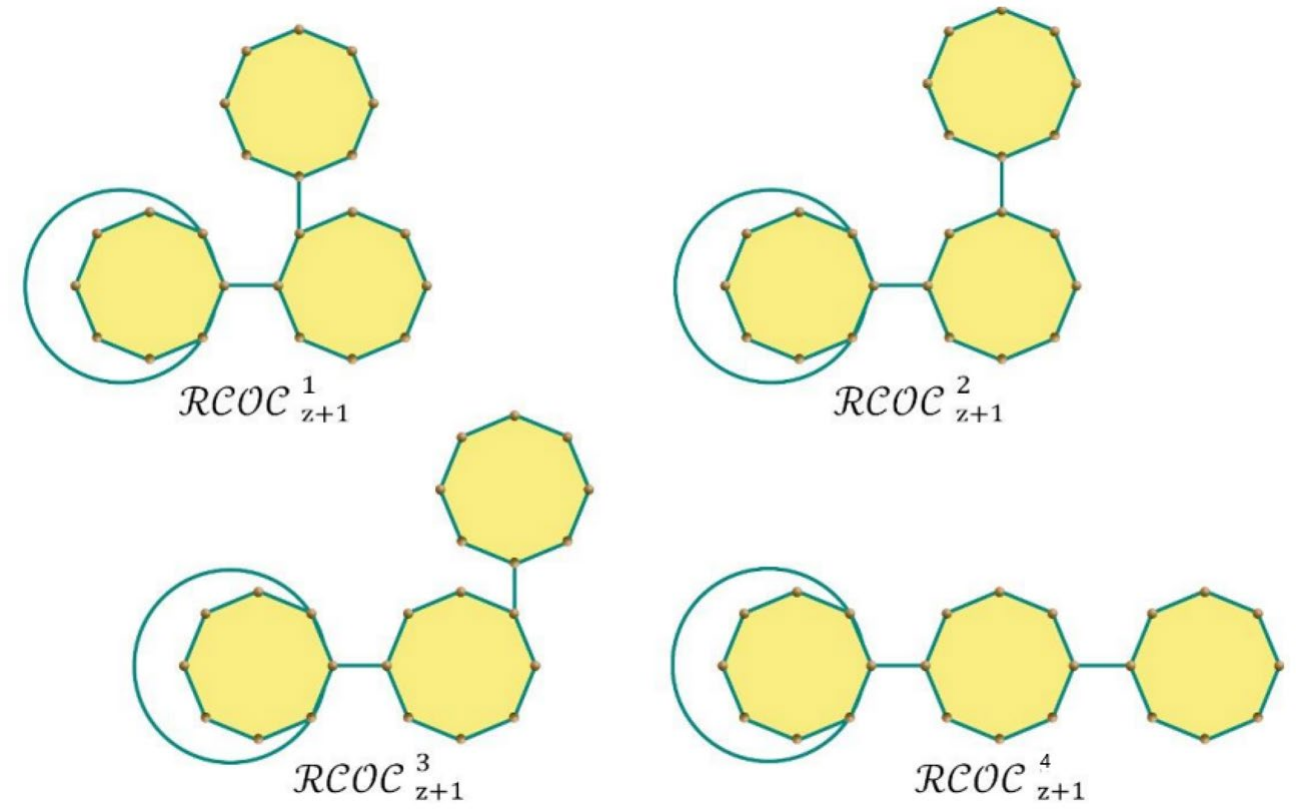
Four types of local arrangements in cyclooctane chains for $z > 3.$

The topological indices of the molecular structures that have been derived from their corresponding chemical graphs are called the molecular descriptors. There are many topological indices that have some applications in structural chemistry, especially in QSPR/QSAR research. The cyclooctanes have distinctive physicochemical properties due to saturated and unsaturated hydrocarbons. The cyclooctane chains are made up of a specific arrangement of eight-membered rings. A random cyclooctane chain is an arrangement of octagons such that any two consecutive octagons are attached by an edge in a random way. We use the notation $\mathcal{R}\mathcal{C}\mathcal{O}\mathcal{C}$_m_ to denote a cyclooctane chain with m number of octagons. For ${\mathcalligra{z}}=1, 2,$ Fig. 1 represents the unique arrangement in $\mathcal{R}\mathcal{C}\mathcal{O}\mathcal{C}$_m_. For ${\mathcalligra{z}}=3$, we get four $\mathcal{R}\mathcal{C}\mathcal{O}\mathcal{C}$_m_ chains as shown in Fig. 2. The four types of cyclooctane chains are denoted by ${\mathcal{R}\mathcal{C}\mathcal{O}\mathcal{C}}_{z+1 }^{1}$, ${\mathcal{R}\mathcal{C}\mathcal{O}\mathcal{C}}_{z+1 }^{2}$, ${\mathcal{R}\mathcal{C}\mathcal{O}\mathcal{C}}_{z+1 }^{3}$ and ${\mathcal{R}\mathcal{C}\mathcal{O}\mathcal{C}}_{z+1 }^{4}$(see Fig. 3). Therefore, $\mathcal{R}\mathcal{C}\mathcal{O}\mathcal{C}$ (m; ρ_1_, ρ_2_, ρ_3_) can be attained by stepwise addition of a terminal octagon. The possible four structures that can be made at each step from a random selection (z = 3, 4,…,k) are

(i) $${\mathcal{R}\mathcal{C}\mathcal{O}\mathcal{C}}_{z-1}$$→ $${\mathcal{R}\mathcal{C}\mathcal{O}\mathcal{C}}_{z}^{1}$$ with probability $${m}_{1}$$,
(ii) ${\mathcal{R}\mathcal{C}\mathcal{O}\mathcal{C}}_{z-1}$→${\mathcal{R}\mathcal{C}\mathcal{O}\mathcal{C}}_{z}^{2}$ with probability ${m}_{2}$,
(iii) ${\mathcal{R}\mathcal{C}\mathcal{O}\mathcal{C}}_{z-1}$→${\mathcal{R}\mathcal{C}\mathcal{O}\mathcal{C}}_{z}^{3}$ with probability ${m}_{3}$,
(iv) ${\mathcal{R}\mathcal{C}\mathcal{O}\mathcal{C}}_{z-1}$→ ${\mathcal{R}\mathcal{C}\mathcal{O}\mathcal{C}}_{z}^{4}$ with probability $r=1-{m}_{1}{-m}_{2}-{m}_{3}$

If we assume that the probabilities are constant and independent of the step parameter, then this process is a zeroth-order Markov process. In order to compute the atom-bond connectivity index, the arithmetic–geometric, geometric-arithmetic, and forgotten index of ${\mathcal{R}\mathcal{C}\mathcal{O}\mathcal{C}}_{z}$, we need to find the edge partition of ${\mathcal{R}\mathcal{C}\mathcal{O}\mathcal{C}}_{z}$ depending on the degree of end vertices of each edge. It is easy to see that it contains only (2, 2), (2, 3), and (3, 3)-types of edges. Therefore, the mathematical expression of $\mathcal{A}\mathcal{B}\mathcal{C}$, $\mathcal{G}\mathcal{A}$, $\mathcal{A}\mathcal{G}$, and $\mathcal{F}$ indices can be written as:

5 $$\mathcal{F}\left({\mathcal{R}\mathcal{C}\mathcal{O}\mathcal{C}}_{z}\right)=8{x}_{22}\left({\mathcal{R}\mathcal{C}\mathcal{O}\mathcal{C}}_{z}\right)+13{x}_{23}\left({\mathcal{R}\mathcal{C}\mathcal{O}\mathcal{C}}_{z}\right)+18{x}_{33}\left({\mathcal{R}\mathcal{C}\mathcal{O}\mathcal{C}}_{z}\right)$$

6 $$\mathcal{A}\mathcal{B}\mathcal{C}\left({\mathcal{R}\mathcal{C}\mathcal{O}\mathcal{C}}_{z}\right)=\frac{1}{\sqrt{2}}{x}_{22}\left({\mathcal{R}\mathcal{C}\mathcal{O}\mathcal{C}}_{z}\right)+\frac{1}{\sqrt{2}}{x}_{23}\left({\mathcal{R}\mathcal{C}\mathcal{O}\mathcal{C}}_{z}\right)+\frac{2}{3}{x}_{33}({\mathcal{R}\mathcal{C}\mathcal{O}\mathcal{C}}_{z}).$$

7 $$\mathcal{G}\mathcal{A}\left({\mathcal{R}\mathcal{C}\mathcal{O}\mathcal{C}}_{z}\right)={x}_{22}\left({\mathcal{R}\mathcal{C}\mathcal{O}\mathcal{C}}_{z}\right)+\frac{2\sqrt{6}}{5}{x}_{23}\left({\mathcal{R}\mathcal{C}\mathcal{O}\mathcal{C}}_{z}\right)+{x}_{33}({\mathcal{R}\mathcal{C}\mathcal{O}\mathcal{C}}_{z}).$$

8 $$\mathcal{A}\mathcal{G}\left({\mathcal{R}\mathcal{C}\mathcal{O}\mathcal{C}}_{z}\right)={x}_{22}\left({\mathcal{R}\mathcal{C}\mathcal{O}\mathcal{C}}_{z}\right)+\frac{5}{2\sqrt{6}}{x}_{23}\left({\mathcal{R}\mathcal{C}\mathcal{O}\mathcal{C}}_{z}\right)+{x}_{33}({\mathcal{R}\mathcal{C}\mathcal{O}\mathcal{C}}_{z}).$$

## Results

A random cyclooctane chain ${\mathcal{R}\mathcal{C}\mathcal{O}\mathcal{C}}_{z}$ is a local arrangement. Hence, $\mathcal{F}(\mathcal{R}\mathcal{C}\mathcal{O}\mathcal{C}\left(z;{m}_{1},{m}_{2},{m}_{3})\right)$, $\mathcal{A}\mathcal{B}\mathcal{C}(\mathcal{R}\mathcal{C}\mathcal{O}\mathcal{C}\left(z;{m}_{1},{m}_{2},{m}_{3})\right)$, $\mathcal{G}\mathcal{A}(\mathcal{R}\mathcal{C}\mathcal{O}\mathcal{C}\left(z;{m}_{1},{m}_{2},{m}_{3})\right)$, and $\mathcal{A}\mathcal{G}(\mathcal{R}\mathcal{C}\mathcal{O}\mathcal{C}\left(z;{m}_{1},{m}_{2},{m}_{3})\right)$ are the random variables. We use the notation ${E}_{z}^{\mathcal{F}}=E[\mathcal{F}(\mathcal{R}\mathcal{C}\mathcal{O}\mathcal{C}\left(z;{m}_{1},{m}_{2},{m}_{3})\right)]$, ${E}_{z}^{ABC}=E[\mathcal{A}\mathcal{B}\mathcal{C}(\mathcal{R}\mathcal{C}\mathcal{O}\mathcal{C}\left(z;{m}_{1},{m}_{2},{m}_{3})\right)]$ ,${E}_{z}^{\mathcal{G}\mathcal{A}}=E[\mathcal{G}\mathcal{A}(\mathcal{R}\mathcal{C}\mathcal{O}\mathcal{C}\left(z;{m}_{1},{m}_{2},{m}_{3})\right)]$, and ${E}_{z}^{\mathcal{A}\mathcal{G}}=E[\mathcal{A}\mathcal{G}(\mathcal{R}\mathcal{C}\mathcal{O}\mathcal{C}\left(z;{m}_{1},{m}_{2},{m}_{3})\right)]$ to denote the expected values of the forgotten, the atom bond connectivity, the geometric arithmetic and the arithmetic geometric indices of ${\mathcal{R}\mathcal{C}\mathcal{O}\mathcal{C}}_{z}$ respectively.

### Theorem 1

*Let* $\mathcal{R}\mathcal{C}\mathcal{O}\mathcal{C}\left(z;{m}_{1},{m}_{2},{m}_{3}\right)$* be the random cyclooctane and* $z\ge 2$.* Then*

$${E}_{z}^{\mathcal{F}}=z\left[102\right]-38$$

### Proof

It is easy to see that ${E}_{2}^{\mathcal{F}}=166$, which is indeed true. For z $\ge 3,$ there are four possibilities:

(i) If ${\mathcal{R}\mathcal{C}\mathcal{O}\mathcal{C}}_{z-1}\to {\mathcal{R}\mathcal{C}\mathcal{O}\mathcal{C}}_{z}^{1}$ with probability ${m}_{1}$, then
  $${x}_{22}\left({\mathcal{R}\mathcal{C}\mathcal{O}\mathcal{C}}_{z}^{1}\right)={x}_{22}\left({\mathcal{R}\mathcal{C}\mathcal{O}\mathcal{C}}_{z-1}\right)+5$$, $${x}_{23}\left({\mathcal{R}\mathcal{C}\mathcal{O}\mathcal{C}}_{z}^{1}\right)={x}_{23}\left({\mathcal{R}\mathcal{C}\mathcal{O}\mathcal{C}}_{z-1}\right)+2$$, $${x}_{33}\left(\mathcal{R}\mathcal{C}\mathcal{O}\mathcal{C}\right)={x}_{33}\left({\mathcal{R}\mathcal{C}\mathcal{O}\mathcal{C}}_{z-1}\right)+2$$,
  From Eq. (5), we have $\mathcal{F}\left({\mathcal{R}\mathcal{C}\mathcal{O}\mathcal{C}}_{z}^{1}\right)=\mathcal{F}\left({\mathcal{R}\mathcal{C}\mathcal{O}\mathcal{C}}_{z-1}\right)+102$.
(ii) If ${\mathcal{R}\mathcal{C}\mathcal{O}\mathcal{C}}_{z-1}\to {\mathcal{R}\mathcal{C}\mathcal{O}\mathcal{C}}_{z}^{2}$ with probability ${m}_{2}$, then
  $${x}_{22}\left({\mathcal{R}\mathcal{C}\mathcal{O}\mathcal{C}}_{z}^{2}\right)={x}_{22}\left({\mathcal{R}\mathcal{C}\mathcal{O}\mathcal{C}}_{z-1}\right)+4$$, $${x}_{23}\left({\mathcal{R}\mathcal{C}\mathcal{O}\mathcal{C}}_{z}^{2}\right)={x}_{23}\left({\mathcal{R}\mathcal{C}\mathcal{O}\mathcal{C}}_{z-1}\right)+4$$, $${x}_{33}\left({\mathcal{R}\mathcal{C}\mathcal{O}\mathcal{C}}_{z}^{2}\right)={x}_{33}\left({\mathcal{R}\mathcal{C}\mathcal{O}\mathcal{C}}_{z-1}\right)+1$$,
  From Eq. (5), we have $\mathcal{F}\left({\mathcal{R}\mathcal{C}\mathcal{O}\mathcal{C}}_{z}^{2}\right)=\mathcal{F}\left({\mathcal{R}\mathcal{C}\mathcal{O}\mathcal{C}}_{z-1}\right)+102$.
(iii) If ${\mathcal{R}\mathcal{C}\mathcal{O}\mathcal{C}}_{z-1}\to {\mathcal{R}\mathcal{C}\mathcal{O}\mathcal{C}}_{z}^{3}$ with probability ${m}_{3}$, then
  $${x}_{22}\left({\mathcal{R}\mathcal{C}\mathcal{O}\mathcal{C}}_{z}^{3}\right)={x}_{22}\left({\mathcal{R}\mathcal{C}\mathcal{O}\mathcal{C}}_{z-1}\right)+4$$, $${x}_{23}\left({\mathcal{R}\mathcal{C}\mathcal{O}\mathcal{C}}_{z}^{3}\right)={x}_{23}\left({\mathcal{R}\mathcal{C}\mathcal{O}\mathcal{C}}_{z-1}\right)+4$$, $${x}_{33}\left({\mathcal{R}\mathcal{C}\mathcal{O}\mathcal{C}}_{z}^{3}\right)={x}_{33}\left({\mathcal{R}\mathcal{C}\mathcal{O}\mathcal{C}}_{z-1}\right)+1$$,
  From Eq. (5), we have $\mathcal{F}\left({\mathcal{R}\mathcal{C}\mathcal{O}\mathcal{C}}_{z}^{3}\right)=\mathcal{F}\left({\mathcal{R}\mathcal{C}\mathcal{O}\mathcal{C}}_{z-1}\right)+102$.
(iv) If ${\mathcal{R}\mathcal{C}\mathcal{O}\mathcal{C}}_{z-1}\to {\mathcal{R}\mathcal{C}\mathcal{O}\mathcal{C}}_{z}^{4}$ with probability $1-{m}_{1}-{m}_{2}-{m}_{3}$, then
  $${x}_{22}\left({\mathcal{R}\mathcal{C}\mathcal{O}\mathcal{C}}_{z}^{4}\right)={x}_{22}\left({\mathcal{R}\mathcal{C}\mathcal{O}\mathcal{C}}_{z-1}\right)+4$$, $${x}_{23}\left({\mathcal{R}\mathcal{C}\mathcal{O}\mathcal{C}}_{z}^{4}\right)={x}_{23}\left({\mathcal{R}\mathcal{C}\mathcal{O}\mathcal{C}}_{z-1}\right)+1$$, $${x}_{33}\left({\mathcal{R}\mathcal{C}\mathcal{O}\mathcal{C}}_{z}^{4}\right)={x}_{33}\left({\mathcal{R}\mathcal{C}\mathcal{O}\mathcal{C}}_{z-1}\right)+1$$,

From Eq. (5), we have $\mathcal{F}\left({\mathcal{R}\mathcal{C}\mathcal{O}\mathcal{C}}_{z}^{4}\right)=\mathcal{F}\left({\mathcal{R}\mathcal{C}\mathcal{O}\mathcal{C}}_{z-1}\right)+102$

Thus, we get

$${E}_{z}^{\mathcal{F}}={m}_{1}\mathcal{F}\left({\mathcal{R}\mathcal{C}\mathcal{O}\mathcal{C}}_{z}^{1}\right)+{m}_{2}\mathcal{F}\left({\mathcal{R}\mathcal{C}\mathcal{O}\mathcal{C}}_{z}^{2}\right)+{m}_{3}\mathcal{F}\left({\mathcal{R}\mathcal{C}\mathcal{O}\mathcal{C}}_{z}^{3}\right)+\left(1-{m}_{1}-{m}_{2}-{m}_{3}\right)\mathcal{F}\left({\mathcal{R}\mathcal{C}\mathcal{O}\mathcal{C}}_{z}^{4}\right)$$

$$={m}_{1}\left[\mathcal{F}\left({\mathcal{R}\mathcal{C}\mathcal{O}\mathcal{C}}_{z}+102\right)\right]+{m}_{2}\left[\mathcal{F}\left({\mathcal{R}\mathcal{C}\mathcal{O}\mathcal{C}}_{z}+102\right)\right]+{m}_{3}\left[\mathcal{F}\left({\mathcal{R}\mathcal{C}\mathcal{O}\mathcal{C}}_{z}+102\right)\right]+\left(1-{m}_{1}-{m}_{2}-{m}_{3}\right)\left[\mathcal{F}\left({\mathcal{R}\mathcal{C}\mathcal{O}\mathcal{C}}_{z}+102\right)\right]$$

9 $${E}_{z}^{\mathcal{F}}=\mathcal{F}\left({\mathcal{R}\mathcal{C}\mathcal{O}\mathcal{C}}_{z-1}\right)+102$$

Since ${E[E}_{z}^{\mathcal{F}}]={E}_{z}^{\mathcal{F}}$, by applying operator on Eq. (9), we obtain

10 $${E}_{z}^{\mathcal{F}}={E}_{z-1}^{\mathcal{F}}+102, \quad m>2$$

Using the initial conditions and solving recurrence relation, we get

$${E}_{z}^{\mathcal{F}}=z\left[102\right]-38.$$

### Theorem 2

*Let* $\mathcal{R}\mathcal{C}\mathcal{O}\mathcal{C}\left(z;{m}_{1},{m}_{2},{m}_{3}\right)$* be the random cyclooctane and* $z\ge 2$.* Then*

$${E}_{z}^{\mathcal{A}\mathcal{B}\mathcal{C}}=z\left[\left(\frac{4-3\sqrt{2}}{6}\right){m}_{1}+\frac{2+12\sqrt{2}}{3}\right]-\left(\frac{4-3\sqrt{2}}{3}\right){m}_{1}-\frac{2}{3}$$

### Proof

It is easy to calculate that ${E}_{2}^{\mathcal{A}\mathcal{B}\mathcal{C}}=\frac{2+24\sqrt{2}}{3}$, which is indeed true. For z $\ge 3,$ we have four possibilities:

- If ${\mathcal{R}\mathcal{C}\mathcal{O}\mathcal{C}}_{z-1}\to {\mathcal{R}\mathcal{C}\mathcal{O}\mathcal{C}}_{z}^{1}$ with probability ${m}_{1}$, then
  $${x}_{22}\left({\mathcal{R}\mathcal{C}\mathcal{O}\mathcal{C}}_{z}^{1}\right)={x}_{22}\left({\mathcal{R}\mathcal{C}\mathcal{O}\mathcal{C}}_{z-1}\right)+5$$, $${x}_{23}\left({\mathcal{R}\mathcal{C}\mathcal{O}\mathcal{C}}_{z}^{1}\right)={x}_{23}\left({\mathcal{R}\mathcal{C}\mathcal{O}\mathcal{C}}_{z-1}\right)+2$$, $${x}_{33}\left({\mathcal{R}\mathcal{C}\mathcal{O}\mathcal{C}}_{z}^{1}\right)={x}_{33}\left({\mathcal{R}\mathcal{C}\mathcal{O}\mathcal{C}}_{z-1}\right)+2$$,
  From Eq. (6), we have $\mathcal{A}\mathcal{B}\mathcal{C}\left({\mathcal{R}\mathcal{C}\mathcal{O}\mathcal{C}}_{z}^{1}\right)=\mathcal{A}\mathcal{B}\mathcal{C}\left({\mathcal{R}\mathcal{C}\mathcal{O}\mathcal{C}}_{z-1}\right)+\frac{8+21\sqrt{2}}{6}$.
- If ${\mathcal{R}\mathcal{C}\mathcal{O}\mathcal{C}}_{z-1}\to {\mathcal{R}\mathcal{C}\mathcal{O}\mathcal{C}}_{z}^{2}$ with probability ${m}_{2}$, then
  $${x}_{22}\left({\mathcal{R}\mathcal{C}\mathcal{O}\mathcal{C}}_{z}^{2}\right)={x}_{22}\left({\mathcal{R}\mathcal{C}\mathcal{O}\mathcal{C}}_{z-1}\right)+4$$, $${x}_{23}\left({\mathcal{R}\mathcal{C}\mathcal{O}\mathcal{C}}_{z}^{2}\right)={x}_{23}\left({R\mathcal{R}\mathcal{C}\mathcal{O}\mathcal{C}}_{z-1}\right)+4$$, $${x}_{33}\left({\mathcal{R}\mathcal{C}\mathcal{O}\mathcal{C}}_{z}^{2}\right)={x}_{33}\left({\mathcal{R}\mathcal{C}\mathcal{O}\mathcal{C}}_{z-1}\right)+1$$,
  From Eq. (6), we have $\mathcal{A}\mathcal{B}\mathcal{C}\left({\mathcal{R}\mathcal{C}\mathcal{O}\mathcal{C}}_{z}^{2}\right)=\mathcal{A}\mathcal{B}\mathcal{C}\left({\mathcal{R}\mathcal{C}\mathcal{O}\mathcal{C}}_{z-1}\right)+\frac{2+12\sqrt{2}}{3}.$
- If ${\mathcal{R}\mathcal{C}\mathcal{O}\mathcal{C}}_{z-1}\to {\mathcal{R}\mathcal{C}\mathcal{O}\mathcal{C}}_{z}^{3}$ with probability ${m}_{3}$, then
  $${x}_{22}\left({\mathcal{R}\mathcal{C}\mathcal{O}\mathcal{C}}_{z}^{3}\right)={x}_{22}\left({\mathcal{R}\mathcal{C}\mathcal{O}\mathcal{C}}_{z-1}\right)+4$$, $${x}_{23}\left({\mathcal{R}\mathcal{C}\mathcal{O}\mathcal{C}}_{z}^{3}\right)={x}_{23}\left({\mathcal{R}\mathcal{C}\mathcal{O}\mathcal{C}}_{z-1}\right)+4$$, $${x}_{33}\left({\mathcal{R}\mathcal{C}\mathcal{O}\mathcal{C}}_{z}^{3}\right)={x}_{33}\left({\mathcal{R}\mathcal{C}\mathcal{O}\mathcal{C}}_{z-1}\right)+1$$,
  From Eq. (6), we have $\mathcal{A}\mathcal{B}\mathcal{C}\left({\mathcal{R}\mathcal{C}\mathcal{O}\mathcal{C}}_{z}^{3}\right)=\mathcal{A}\mathcal{B}\mathcal{C}\left({\mathcal{R}\mathcal{C}\mathcal{O}\mathcal{C}}_{z-1}\right)+\frac{2+12\sqrt{2}}{3}$.
- If ${\mathcal{R}\mathcal{C}\mathcal{O}\mathcal{C}}_{z-1}\to {\mathcal{R}\mathcal{C}\mathcal{O}\mathcal{C}}_{z}^{4}$ with probability $1-{m}_{1}-{m}_{2}-{m}_{3}$, then
  $${x}_{22}\left({\mathcal{R}\mathcal{C}\mathcal{O}\mathcal{C}}_{z}^{4}\right)={x}_{22}\left({\mathcal{R}\mathcal{C}\mathcal{O}\mathcal{C}}_{z-1}\right)+4$$, $${x}_{23}\left({\mathcal{R}\mathcal{C}\mathcal{O}\mathcal{C}}_{z}^{4}\right)={x}_{23}\left({\mathcal{R}\mathcal{C}\mathcal{O}\mathcal{C}}_{z-1}\right)+1$$, $${x}_{33}\left({\mathcal{R}\mathcal{C}\mathcal{O}\mathcal{C}}_{z}^{4}\right)={x}_{33}\left({\mathcal{R}\mathcal{C}\mathcal{O}\mathcal{C}}_{z-1}\right)+1$$,
  From Eq. (6), we have $\mathcal{A}\mathcal{B}\mathcal{C}\left({\mathcal{R}\mathcal{C}\mathcal{O}\mathcal{C}}_{z}^{4}\right)=\mathcal{A}\mathcal{B}\mathcal{C}\left({\mathcal{R}\mathcal{C}\mathcal{O}\mathcal{C}}_{z-1}\right)+\frac{2+12\sqrt{2}}{3}$.

Thus, we get

$${E}_{z}^{\mathcal{A}\mathcal{B}\mathcal{C}}={m}_{1}\mathcal{A}\mathcal{B}\mathcal{C}\left({\mathcal{R}\mathcal{C}\mathcal{O}\mathcal{C}}_{z}^{1}\right)+{m}_{2}\mathcal{A}\mathcal{B}\mathcal{C}\left({\mathcal{R}\mathcal{C}\mathcal{O}\mathcal{C}}_{z}^{2}\right)+{m}_{3}\mathcal{A}\mathcal{B}\mathcal{C}\left({\mathcal{R}\mathcal{C}\mathcal{O}\mathcal{C}}_{z}^{3}\right)+\left(1-{m}_{1}-{m}_{2}-{m}_{3}\right)\mathcal{A}\mathcal{B}\mathcal{C}\left({\mathcal{R}\mathcal{C}\mathcal{O}\mathcal{C}}_{z}^{4}\right)$$

$$={m}_{1}\left[\mathcal{A}\mathcal{B}\mathcal{C}\left({\mathcal{R}\mathcal{C}\mathcal{O}\mathcal{C}}_{z}+\frac{8+21\sqrt{2}}{6}\right)\right]+{m}_{2}\left[\mathcal{A}\mathcal{B}\mathcal{C}\left({\mathcal{R}\mathcal{C}\mathcal{O}\mathcal{C}}_{z}+\frac{2+12\sqrt{2}}{3}\right)\right]+{m}_{3}\left[\mathcal{A}\mathcal{B}\mathcal{C}\left({\mathcal{R}\mathcal{C}\mathcal{O}\mathcal{C}}_{z}+\frac{2+12\sqrt{2}}{3}\right)\right]+\left(1-{m}_{1}-{m}_{2}-{m}_{3}\right)\left[\mathcal{A}\mathcal{B}\mathcal{C}\left({\mathcal{R}\mathcal{C}\mathcal{O}\mathcal{C}}_{z}+\frac{2+12\sqrt{2}}{3}\right)\right]$$

11 $${E}_{z}^{\mathcal{A}\mathcal{B}\mathcal{C}}=\mathcal{A}\mathcal{B}\mathcal{C}\left({\mathcal{R}\mathcal{C}\mathcal{O}\mathcal{C}}_{z-1}\right)+\left(\frac{4-3\sqrt{2}}{6}\right){m}_{1}+\frac{2+12\sqrt{2}}{3}$$

Since ${E[E}_{z}^{\mathcal{A}\mathcal{B}\mathcal{C}}]={E}_{z}^{\mathcal{A}\mathcal{B}\mathcal{C}}$, by applying operator on Eq. (11), we obtain

12 $${E}_{z}^{\mathcal{A}\mathcal{B}\mathcal{C}}={E}_{z-1}^{\mathcal{A}\mathcal{B}\mathcal{C}}+\left(\frac{4-3\sqrt{2}}{6}\right){m}_{1}+\frac{2+12\sqrt{2}}{3}, m>2$$

Using the initial conditions and solving the recurrence relation, we get

$${E}_{z}^{\mathcal{A}\mathcal{B}\mathcal{C}}=z\left[\left(\frac{4-3\sqrt{2}}{6}\right){m}_{1}+\frac{2+12\sqrt{2}}{3}\right]-\left(\frac{4-3\sqrt{2}}{3}\right){m}_{1}-\frac{2}{3}.$$

### Theorem 3

*Let* $\mathcal{R}\mathcal{C}\mathcal{O}\mathcal{C}\left(z;{m}_{1},{m}_{2},{m}_{3}\right)$* be the random cyclooctane and* $z\ge 2$.* Then*

$${E}_{z}^{\mathcal{G}\mathcal{A}}=z\left[\left(\frac{10-4\sqrt{6}}{5}\right){m}_{1}+\frac{25+8\sqrt{6}}{5}\right]-\left(\frac{20-8\sqrt{6}}{5}\right){m}_{1}+\frac{15-8\sqrt{6}}{5}$$

### Proof

Observe that ${E}_{2}^{\mathcal{G}\mathcal{A}}=\frac{65+8\sqrt{6}}{5}$ , which is indeed true.. For z $\ge 3,$ we have four possibilities:

- If ${\mathcal{R}\mathcal{C}\mathcal{O}\mathcal{C}}_{z-1}\to {\mathcal{R}\mathcal{C}\mathcal{O}\mathcal{C}}_{z}^{1}$ with probability ${m}_{1}$, then
  $${x}_{22}\left({\mathcal{R}\mathcal{C}\mathcal{O}\mathcal{C}}_{z}^{1}\right)={x}_{22}\left({\mathcal{R}\mathcal{C}\mathcal{O}\mathcal{C}}_{z-1}\right)+5$$, $${x}_{23}\left({\mathcal{R}\mathcal{C}\mathcal{O}\mathcal{C}}_{z}^{1}\right)={x}_{23}\left({\mathcal{R}\mathcal{C}\mathcal{O}\mathcal{C}}_{z-1}\right)+2$$, $${x}_{33}\left({\mathcal{R}\mathcal{C}\mathcal{O}\mathcal{C}}_{z}^{1}\right)={x}_{33}\left({\mathcal{R}\mathcal{C}\mathcal{O}\mathcal{C}}_{z-1}\right)+2$$,
  From Eq. (7), we have $\mathcal{G}\mathcal{A}\left({\mathcal{R}\mathcal{C}\mathcal{O}\mathcal{C}}_{z}^{1}\right)=\mathcal{G}\mathcal{A}\left({\mathcal{R}\mathcal{C}\mathcal{O}\mathcal{C}}_{z-1}\right)+\frac{35+4\sqrt{6}}{5}$.
- If ${\mathcal{R}\mathcal{C}\mathcal{O}\mathcal{C}}_{z-1}\to {\mathcal{R}\mathcal{C}\mathcal{O}\mathcal{C}}_{z}^{2}$ with probability ${m}_{2}$, then
  $${x}_{22}\left({\mathcal{R}\mathcal{C}\mathcal{O}\mathcal{C}}_{z}^{2}\right)={x}_{22}\left({\mathcal{R}\mathcal{C}\mathcal{O}\mathcal{C}}_{z-1}\right)+4$$, $${x}_{23}\left({\mathcal{R}\mathcal{C}\mathcal{O}\mathcal{C}}_{z}^{2}\right)={x}_{23}\left({R\mathcal{R}\mathcal{C}\mathcal{O}\mathcal{C}}_{z-1}\right)+4$$, $${x}_{33}\left({\mathcal{R}\mathcal{C}\mathcal{O}\mathcal{C}}_{z}^{2}\right)={x}_{33}\left({\mathcal{R}\mathcal{C}\mathcal{O}\mathcal{C}}_{z-1}\right)+1$$,
  From Eq. (7), we have $\mathcal{G}\mathcal{A}\left({\mathcal{R}\mathcal{C}\mathcal{O}\mathcal{C}}_{z}^{2}\right)=\mathcal{G}\mathcal{A}\left({\mathcal{R}\mathcal{C}\mathcal{O}\mathcal{C}}_{z-1}\right)+\frac{25+8\sqrt{6}}{5}.$

(iii) If ${\mathcal{R}\mathcal{C}\mathcal{O}\mathcal{C}}_{z-1}\to {\mathcal{R}\mathcal{C}\mathcal{O}\mathcal{C}}_{z}^{3}$ with probability ${m}_{3}$, then
  $${x}_{22}\left({\mathcal{R}\mathcal{C}\mathcal{O}\mathcal{C}}_{z}^{3}\right)={x}_{22}\left({\mathcal{R}\mathcal{C}\mathcal{O}\mathcal{C}}_{z-1}\right)+4$$, $${x}_{23}\left({\mathcal{R}\mathcal{C}\mathcal{O}\mathcal{C}}_{z}^{3}\right)={x}_{23}\left({\mathcal{R}\mathcal{C}\mathcal{O}\mathcal{C}}_{z-1}\right)+4$$, $${x}_{33}\left({\mathcal{R}\mathcal{C}\mathcal{O}\mathcal{C}}_{z}^{3}\right)={x}_{33}\left({\mathcal{R}\mathcal{C}\mathcal{O}\mathcal{C}}_{z-1}\right)+1$$,
  From Eq. (7), we have $\mathcal{G}\mathcal{A}\left({\mathcal{R}\mathcal{C}\mathcal{O}\mathcal{C}}_{z}^{3}\right)=\mathcal{G}\mathcal{A}\left({\mathcal{R}\mathcal{C}\mathcal{O}\mathcal{C}}_{z-1}\right)+\frac{25+8\sqrt{6}}{5}$.
(iv) If ${\mathcal{R}\mathcal{C}\mathcal{O}\mathcal{C}}_{z-1}\to {\mathcal{R}\mathcal{C}\mathcal{O}\mathcal{C}}_{z}^{4}$ with probability $1-{m}_{1}-{m}_{2}-{m}_{3}$, then
  $${x}_{22}\left({\mathcal{R}\mathcal{C}\mathcal{O}\mathcal{C}}_{z}^{4}\right)={x}_{22}\left({\mathcal{R}\mathcal{C}\mathcal{O}\mathcal{C}}_{z-1}\right)+4$$, $${x}_{23}\left({\mathcal{R}\mathcal{C}\mathcal{O}\mathcal{C}}_{z}^{4}\right)={x}_{23}\left({\mathcal{R}\mathcal{C}\mathcal{O}\mathcal{C}}_{z-1}\right)+1$$, $${x}_{33}\left({\mathcal{R}\mathcal{C}\mathcal{O}\mathcal{C}}_{z}^{4}\right)={x}_{33}\left({\mathcal{R}\mathcal{C}\mathcal{O}\mathcal{C}}_{z-1}\right)+1$$,
  From Eq. (7), we have $\mathcal{G}\mathcal{A}\left({\mathcal{R}\mathcal{C}\mathcal{O}\mathcal{C}}_{z}^{4}\right)=\mathcal{G}\mathcal{A}\left({\mathcal{R}\mathcal{C}\mathcal{O}\mathcal{C}}_{z-1}\right)+\frac{25+8\sqrt{6}}{5}$.

Thus, we get

$${E}_{z}^{\mathcal{G}\mathcal{A}}={m}_{1}\mathcal{G}\mathcal{A}\left({\mathcal{R}\mathcal{C}\mathcal{O}\mathcal{C}}_{z}^{1}\right)+{m}_{2}\mathcal{G}\mathcal{A}\left({\mathcal{R}\mathcal{C}\mathcal{O}\mathcal{C}}_{z}^{2}\right)+{m}_{3}\mathcal{G}\mathcal{A}\left({\mathcal{R}\mathcal{C}\mathcal{O}\mathcal{C}}_{z}^{3}\right)+\left(1-{m}_{1}-{m}_{2}-{m}_{3}\right)\mathcal{G}\mathcal{A}\left({\mathcal{R}\mathcal{C}\mathcal{O}\mathcal{C}}_{z}^{4}\right)$$

$$={m}_{1}\left[\mathcal{G}\mathcal{A}\left({\mathcal{R}\mathcal{C}\mathcal{O}\mathcal{C}}_{z}+\frac{35+4\sqrt{6}}{5}\right)\right]+{m}_{2}\left[\mathcal{G}\mathcal{A}\left({\mathcal{R}\mathcal{C}\mathcal{O}\mathcal{C}}_{z}+\frac{25+8\sqrt{6}}{5}\right)\right]+{m}_{3}\left[\mathcal{G}\mathcal{A}\left({\mathcal{R}\mathcal{C}\mathcal{O}\mathcal{C}}_{z}+\frac{25+8\sqrt{6}}{5}\right)\right]+\left(1-{m}_{1}-{m}_{2}-{m}_{3}\right)\left[\mathcal{G}\mathcal{A}\left({\mathcal{R}\mathcal{C}\mathcal{O}\mathcal{C}}_{z}+\frac{25+8\sqrt{6}}{5}\right)\right]$$

13 $${E}_{z}^{\mathcal{G}\mathcal{A}}=\mathcal{G}\mathcal{A}\left({\mathcal{R}\mathcal{C}\mathcal{O}\mathcal{C}}_{z-1}\right)+\left(\frac{10-4\sqrt{6}}{5}\right){m}_{1}+\frac{25+8\sqrt{6}}{5}$$

Since ${E[E}_{z}^{\mathcal{G}\mathcal{A}}]={E}_{z}^{\mathcal{G}\mathcal{A}}$, by applying operator on Eq. (13), we obtain

14 $${E}_{z}^{\mathcal{G}\mathcal{A}}={E}_{z-1}^{\mathcal{G}\mathcal{A}}+\left(\frac{10-4\sqrt{6}}{5}\right){m}_{1}+\frac{25+8\sqrt{6}}{5}, m>2$$

Using the initial conditions and solving recurrence relation, we get

$${E}_{z}^{\mathcal{G}\mathcal{A}}=z\left[\left(\frac{10-4\sqrt{6}}{5}\right){m}_{1}+\frac{25+8\sqrt{6}}{5}\right]-\left(\frac{20-8\sqrt{6}}{5}\right){m}_{1}+\frac{15-8\sqrt{6}}{5}$$

### Theorem 4

*Let* $\mathcal{R}\mathcal{C}\mathcal{O}\mathcal{C}\left(z;{m}_{1},{m}_{2},{m}_{3}\right)$* be the random cyclooctane and* $z\ge 2$.* Then*

$${E}_{z}^{\mathcal{A}\mathcal{G}}=z\left[\left(\frac{12-5\sqrt{6}}{6}\right){m}_{1}+\frac{15+5\sqrt{6}}{3}\right]-\left(\frac{12-5\sqrt{6}}{3}\right){m}_{1}+\frac{9-5\sqrt{6}}{3}$$

### Proof

It is easy to see that ${E}_{2}^{\mathcal{A}\mathcal{G}}=\frac{39+5\sqrt{6}}{3}$ which is indeed true. For z $\ge 3$, we have four possibilities:

- If ${\mathcal{R}\mathcal{C}\mathcal{O}\mathcal{C}}_{z-1}\to {\mathcal{R}\mathcal{C}\mathcal{O}\mathcal{C}}_{z}^{1}$ with probability ${m}_{1}$, then
  $${x}_{22}\left({\mathcal{R}\mathcal{C}\mathcal{O}\mathcal{C}}_{z}^{1}\right)={x}_{22}\left({\mathcal{R}\mathcal{C}\mathcal{O}\mathcal{C}}_{z-1}\right)+5$$, $${x}_{23}\left({\mathcal{R}\mathcal{C}\mathcal{O}\mathcal{C}}_{z}^{1}\right)={x}_{23}\left({\mathcal{R}\mathcal{C}\mathcal{O}\mathcal{C}}_{z-1}\right)+2$$, $${x}_{33}\left({\mathcal{R}\mathcal{C}\mathcal{O}\mathcal{C}}_{z}^{1}\right)={x}_{33}\left({\mathcal{R}\mathcal{C}\mathcal{O}\mathcal{C}}_{z-1}\right)+2$$,
  From Eq. (8), we have $\mathcal{A}\mathcal{G}\left({\mathcal{R}\mathcal{C}\mathcal{O}\mathcal{C}}_{z}^{1}\right)=\mathcal{A}\mathcal{G}\left({\mathcal{R}\mathcal{C}\mathcal{O}\mathcal{C}}_{z-1}\right)+\frac{42+5\sqrt{6}}{6}$.
- If ${\mathcal{R}\mathcal{C}\mathcal{O}\mathcal{C}}_{z-1}\to {\mathcal{R}\mathcal{C}\mathcal{O}\mathcal{C}}_{z}^{2}$ with probability ${m}_{2}$, then
  $${x}_{22}\left({\mathcal{R}\mathcal{C}\mathcal{O}\mathcal{C}}_{z}^{2}\right)={x}_{22}\left({\mathcal{R}\mathcal{C}\mathcal{O}\mathcal{C}}_{z-1}\right)+4$$, $${x}_{23}\left({\mathcal{R}\mathcal{C}\mathcal{O}\mathcal{C}}_{z}^{2}\right)={x}_{23}\left({R\mathcal{R}\mathcal{C}\mathcal{O}\mathcal{C}}_{z-1}\right)+4$$, $${x}_{33}\left({\mathcal{R}\mathcal{C}\mathcal{O}\mathcal{C}}_{z}^{2}\right)={x}_{33}\left({\mathcal{R}\mathcal{C}\mathcal{O}\mathcal{C}}_{z-1}\right)+1$$,
  From Eq. (8), we have $\mathcal{A}\mathcal{G}\left({\mathcal{R}\mathcal{C}\mathcal{O}\mathcal{C}}_{z}^{2}\right)=\mathcal{A}\mathcal{G}\left({\mathcal{R}\mathcal{C}\mathcal{O}\mathcal{C}}_{z-1}\right)+\frac{15+5\sqrt{6}}{3}$.
- If ${\mathcal{R}\mathcal{C}\mathcal{O}\mathcal{C}}_{z-1}\to {\mathcal{R}\mathcal{C}\mathcal{O}\mathcal{C}}_{z}^{3}$ with probability ${m}_{3}$, then
  $${x}_{22}\left({\mathcal{R}\mathcal{C}\mathcal{O}\mathcal{C}}_{z}^{3}\right)={x}_{22}\left({\mathcal{R}\mathcal{C}\mathcal{O}\mathcal{C}}_{z-1}\right)+4$$, $${x}_{23}\left({\mathcal{R}\mathcal{C}\mathcal{O}\mathcal{C}}_{z}^{3}\right)={x}_{23}\left({\mathcal{R}\mathcal{C}\mathcal{O}\mathcal{C}}_{z-1}\right)+4$$, $${x}_{33}\left({\mathcal{R}\mathcal{C}\mathcal{O}\mathcal{C}}_{z}^{3}\right)={x}_{33}\left({\mathcal{R}\mathcal{C}\mathcal{O}\mathcal{C}}_{z-1}\right)+1$$,
  From Eq. (8), we have $\mathcal{A}\mathcal{G}\left({\mathcal{R}\mathcal{C}\mathcal{O}\mathcal{C}}_{z}^{3}\right)=\mathcal{A}\mathcal{G}\left({\mathcal{R}\mathcal{C}\mathcal{O}\mathcal{C}}_{z-1}\right)+\frac{15+5\sqrt{6}}{3}$
- If ${\mathcal{R}\mathcal{C}\mathcal{O}\mathcal{C}}_{z-1}\to {\mathcal{R}\mathcal{C}\mathcal{O}\mathcal{C}}_{z}^{4}$ with probability $1-{m}_{1}-{m}_{2}-{m}_{3}$, then
  $${x}_{22}\left({\mathcal{R}\mathcal{C}\mathcal{O}\mathcal{C}}_{z}^{4}\right)={x}_{22}\left({\mathcal{R}\mathcal{C}\mathcal{O}\mathcal{C}}_{z-1}\right)+4$$, $${x}_{23}\left({\mathcal{R}\mathcal{C}\mathcal{O}\mathcal{C}}_{z}^{4}\right)={x}_{23}\left({\mathcal{R}\mathcal{C}\mathcal{O}\mathcal{C}}_{z-1}\right)+1$$, $${x}_{33}\left({\mathcal{R}\mathcal{C}\mathcal{O}\mathcal{C}}_{z}^{4}\right)={x}_{33}\left({\mathcal{R}\mathcal{C}\mathcal{O}\mathcal{C}}_{z-1}\right)+1$$,
  From Eq. (8), we have $\mathcal{A}\mathcal{G}\left({\mathcal{R}\mathcal{C}\mathcal{O}\mathcal{C}}_{z}^{4}\right)=\mathcal{A}\mathcal{G}\left({\mathcal{R}\mathcal{C}\mathcal{O}\mathcal{C}}_{z-1}\right)+\frac{15+5\sqrt{6}}{3}$.

Thus, we get

$${E}_{z}^{\mathcal{A}\mathcal{G}}={m}_{1}\mathcal{A}\mathcal{G}\left({\mathcal{R}\mathcal{C}\mathcal{O}\mathcal{C}}_{z}^{1}\right)+{m}_{2}\mathcal{A}\mathcal{G}\left({\mathcal{R}\mathcal{C}\mathcal{O}\mathcal{C}}_{z}^{2}\right)+{m}_{3}\mathcal{A}\mathcal{G}\left({\mathcal{R}\mathcal{C}\mathcal{O}\mathcal{C}}_{z}^{3}\right)+\left(1-{m}_{1}-{m}_{2}-{m}_{3}\right)\mathcal{A}\mathcal{G}\left({\mathcal{R}\mathcal{C}\mathcal{O}\mathcal{C}}_{z}^{4}\right)$$

$$={m}_{1}\left[\mathcal{A}\mathcal{G}\left({\mathcal{R}\mathcal{C}\mathcal{O}\mathcal{C}}_{z}+\frac{42+5\sqrt{6}}{6}\right)\right]+{m}_{2}\left[\mathcal{A}\mathcal{G}\left({\mathcal{R}\mathcal{C}\mathcal{O}\mathcal{C}}_{z}+\frac{15+5\sqrt{6}}{3}\right)\right]+{m}_{3}\left[\mathcal{A}\mathcal{G}\left({\mathcal{R}\mathcal{C}\mathcal{O}\mathcal{C}}_{z}+\frac{15+5\sqrt{6}}{3}\right)\right]+\left(1-{m}_{1}-{m}_{2}-{m}_{3}\right)\left[\mathcal{A}\mathcal{G}\left({\mathcal{R}\mathcal{C}\mathcal{O}\mathcal{C}}_{z}+\frac{15+5\sqrt{6}}{3}\right)\right]$$

15 $${E}_{z}^{\mathcal{A}\mathcal{G}}=\mathcal{A}\mathcal{G}\left({\mathcal{R}\mathcal{C}\mathcal{O}\mathcal{C}}_{z-1}\right)+\left(\frac{12-5\sqrt{6}}{6}\right){m}_{1}+\frac{15+5\sqrt{6}}{3}$$

Since, ${E[E}_{z}^{\mathcal{A}\mathcal{G}}]={E}_{z}^{\mathcal{A}\mathcal{G}}$, by applying operator on Eq. (15), we obtain

16 $${E}_{z}^{\mathcal{A}\mathcal{G}}={E}_{z-1}^{\mathcal{A}\mathcal{G}}+\left(\frac{12-5\sqrt{6}}{6}\right){m}_{1}+\frac{15+5\sqrt{6}}{3}, m>2$$

Using the initial conditions and solving the recurrence relation, we get

$${E}_{z}^{\mathcal{A}\mathcal{G}}=z\left[\left(\frac{12-5\sqrt{6}}{6}\right){m}_{1}+\frac{15+5\sqrt{6}}{3}\right]-\left(\frac{12-5\sqrt{6}}{3}\right){m}_{1}+\frac{9-5\sqrt{6}}{3}$$

**Figure 4:**
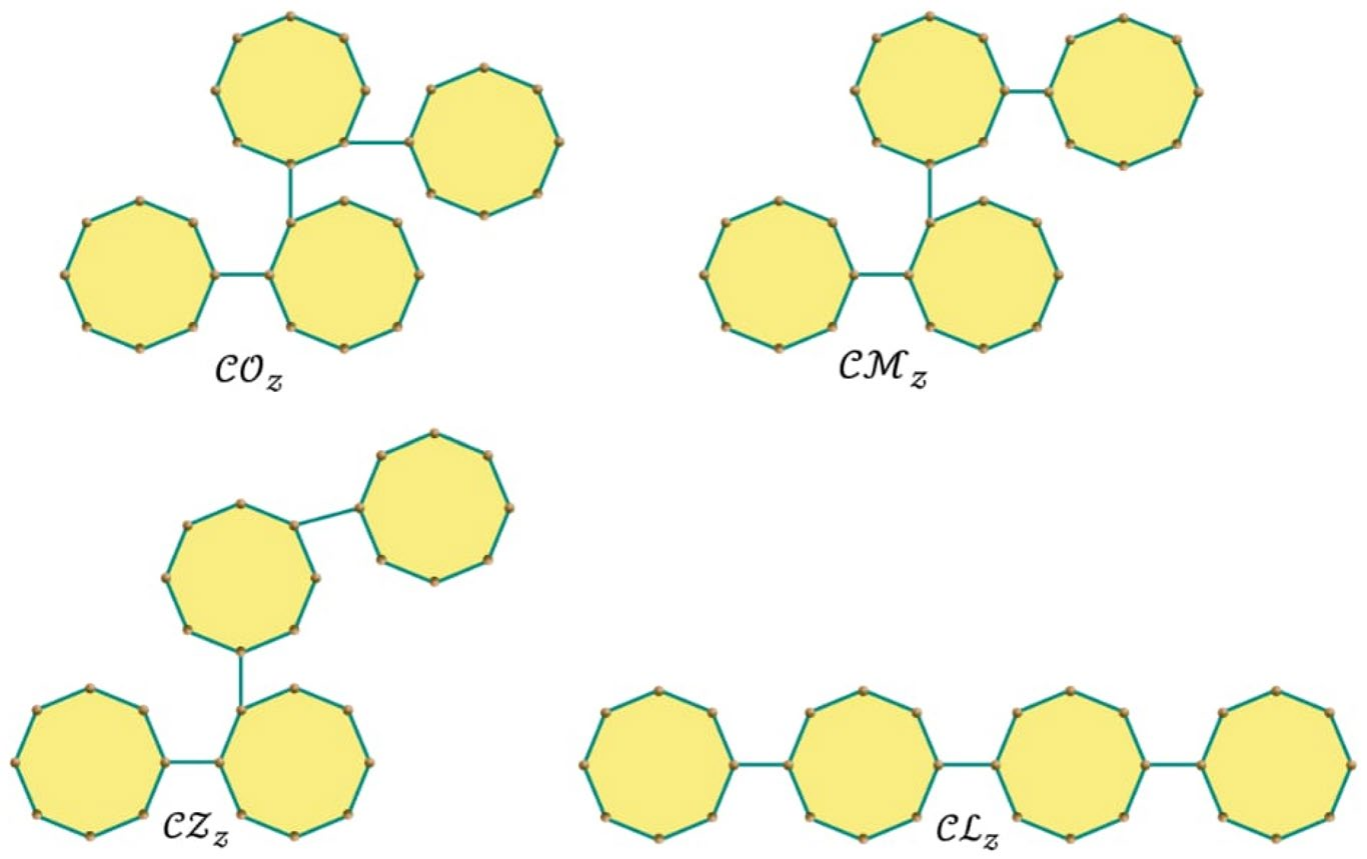
Four special types of cyclooctane chains with ${\mathcalligra{z}}$ octagons.

Now our focus is the special cyclooctanes such as ${\mathcal{C}\mathcal{Z}}_{\mathcalligra{m}}, {\mathcal{C}\mathcal{L}}_{\mathcalligra{m}}, {\mathcal{C}\mathcal{O}}_{\mathcalligra{m}}$, and ${\mathcal{C}\mathcal{M}}_{\mathcalligra{m}}$, which are shown in Fig. 4. Such cyclooctane chains can be obtained as: ${\mathcal{C}\mathcal{Z}}_{\mathcalligra{m}}= \mathcal{R}\mathcal{C}\mathcal{O}\mathcal{C}(m; 1, 0, 0), {\mathcal{C}\mathcal{L}}_{\mathcalligra{m}}=\mathcal{R}\mathcal{C}\mathcal{O}\mathcal{C}(m; 0, 1, 0), {\mathcal{C}\mathcal{O}}_{\mathcalligra{m}}= \mathcal{R}\mathcal{C}\mathcal{O}\mathcal{C}(m; 0, 0, 1),$ and ${\mathcal{C}\mathcal{M}}_{\mathcalligra{m}}= \mathcal{R}\mathcal{C}\mathcal{O}\mathcal{C}(m; 0, 0, 0).$ With the help of Theorems 1–4, we can calculate the expected value of these special type of cyclooctane chains.

### Corollary 1

*For* $m\ge 2$,* we have the following results*:

1. $$\mathcal{F}\left({\mathcal{C}\mathcal{O}}_{\mathcalligra{m}}\right)=102 {z} -38$$  
  $$\mathcal{A}\mathcal{B}\mathcal{C}\left({\mathcal{C}\mathcal{O}}_{\mathcalligra{m}}\right)=\left(\frac{8+21\sqrt{2}}{6}\right){z}-(2-\sqrt{2})$$
  $$\mathcal{G}\mathcal{A}\left({\mathcal{C}\mathcal{O}}_{\mathcalligra{m}}\right)=\left(\frac{35+4\sqrt{6}}{5}\right){z}-1$$
  $$\mathcal{A}\mathcal{G}\left({\mathcal{C}\mathcal{O}}_{\mathcalligra{m}}\right)=\left(\frac{42+5\sqrt{6}}{6}\right) {z}-1$$
2. $$\mathcal{A}\mathcal{B}\mathcal{C}\left({\mathcal{C}\mathcal{M}}_{\mathcalligra{m}}\right)=\mathcal{A}\mathcal{B}\mathcal{C}\left({\mathcal{C}\mathcal{Z}}_{\mathcalligra{m}}\right)=\mathcal{A}\mathcal{B}\mathcal{C}\left({\mathcal{C}\mathcal{L}}_{\mathcalligra{m}}\right)=\left(\frac{2+12\sqrt{2}}{3}\right) {z}-\frac{2}{3}$$
  $$\mathcal{G}\mathcal{A}\left({\mathcal{C}\mathcal{M}}_{\mathcalligra{m}}\right)=\mathcal{G}\mathcal{A}\left({\mathcal{C}\mathcal{Z}}_{\mathcalligra{m}}\right)=\mathcal{G}\mathcal{A}\left({\mathcal{C}\mathcal{L}}_{\mathcalligra{m}}\right)=\left(\frac{25+8\sqrt{6}}{5}\right) {z}+\frac{15-8\sqrt{6}}{5}$$
  $$\mathcal{A}\mathcal{G}\left({\mathcal{C}\mathcal{M}}_{\mathcalligra{m}}\right)=\mathcal{A}\mathcal{G}\left({\mathcal{C}\mathcal{Z}}_{\mathcalligra{m}}\right)=\mathcal{A}\mathcal{G}\left({\mathcal{C}\mathcal{L}}_{\mathcalligra{m}}\right)=\left(\frac{12+5\sqrt{6}}{3}\right) {z}+\frac{9-5\sqrt{6}}{3}$$

## Comparison between the expected values of topological indices

**Figure 5:**
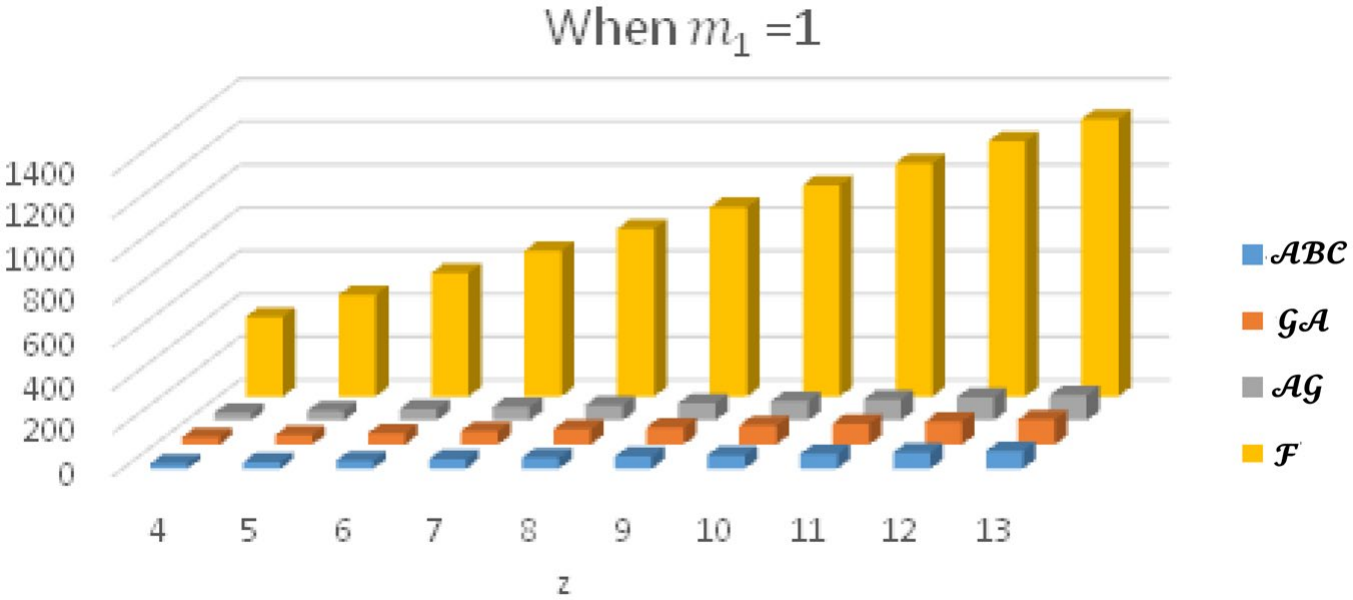
Plot of expected values of topological indices m_1_ = 1.

**Figure 6:**
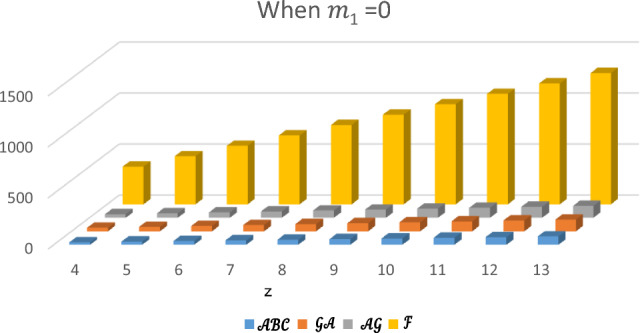
Plot of expected values of topological indices for m_1_ = 0.

**Figure 7:**
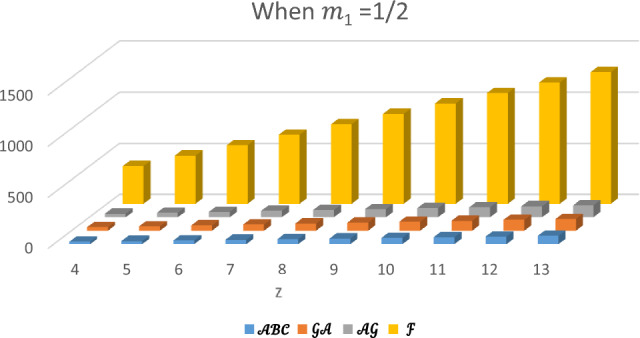
Plot of expected values of topological indices $m_{1} = \frac{1}{2}.$

**Figure 8:**
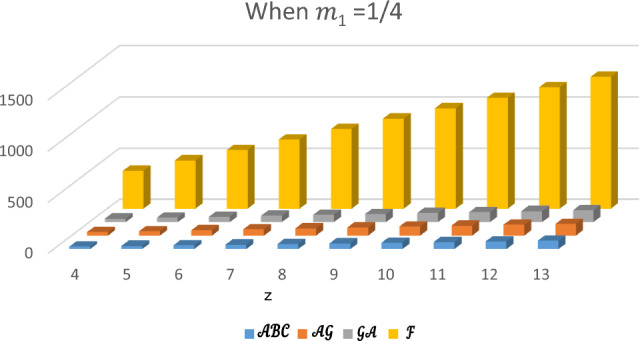
Plot of expected values of topological indices $m_{1} = \frac{1}{4}.$

In this section, the expected values of the forgotten index, the arithmetic–geometric index, the geometric-arithmetic index, and the atom-bond connectivity index are analyzed and compared. In Tables 1, 2, 3 and 4, we have computed the expected values of these indices for different values of ${m}_{1}$. Observe that the value of forgotten index is always greater than the expected values of other topological indices. The graphical comparison between the forgotten index, arithmetic–geometric index, geometric-arithmetic index and atom-bond connectivity index for the cyclooctane chains with the same probabilities is shown in Figs. 5, 6, 7 and 8. One can see that the expected value of the forgotten index is always greater than the expected value of arithmetic–geometric index and the expected value of arithmetic–geometric index is always greater than the expected value of geometric-arithmetic index and the expected value of geometric-arithmetic index is greater than the expected value of atom-bond connectivity index. We give an analytical proof of this fact in the next theorems.

**Table 1:** Expected values of topological indices for ${m}_{1}=1$.

$z$	${E}^{\mathcal{F}}$	${E}^{\mathcal{A}\mathcal{B}\mathcal{C}}$	${E}^{\mathcal{G}\mathcal{A}}$	${E}^{\mathcal{A}\mathcal{G}}$
$4$	$370$	$24.5465$	$34.8388$	$35.1649$
$5$	$472$	$30.8296$	$43.7979$	$44.2062$
$6$	$574$	$37.1126$	$52.7575$	$53.2474$
$7$	$676$	$43.3950$	$61.7171$	$62.2888$
$8$	$778$	$49.6788$	$70.6767$	$71.3299$
$9$	$880$	$55.9615$	$79.6363$	$80.3711$
$10$	$982$	$62.2450$	$88.5959$	$89.4124$
$11$	$1084$	$68.5280$	$97.5555$	$98.4532$
$12$	$1186$	$74.8111$	$106.5150$	$107.4948$
$13$	$1288$	$81.0942$	$115.4746$	$116.5361$

**Table 2:** Expected values of topological indices for ${m}_{1}=0$.

$z$	${E}^{\mathcal{F}}$	${E}^{\mathcal{A}\mathcal{B}\mathcal{C}}$	${E}^{\mathcal{G}\mathcal{A}}$	${E}^{\mathcal{A}\mathcal{G}}$
$4$	$370$	$24.627$	$34.757$	$35.247$
$5$	$472$	$30.950$	$43.676$	$44.329$
$6$	$574$	$37.247$	$52.5959$	$53.412$
$7$	$676$	$43.597$	$61.515$	$62.4948$
$8$	$778$	$49.921$	$70.434$	$71.577$
$9$	$880$	$56.245$	$79.353$	$80659$
$10$	$982$	$62.5685$	$88.272$	$89.6598$
$11$	$1084$	$68.892$	$97.191$	$98.824$
$12$	$1186$	$75.215$	$106.111$	$107.907$
$13$	$1288$	$81.539$	$115.030$	$116.984$

**Table 3:** Expected values of topological indices for ${m}_{1}=\frac{1}{2}$.

$z$	${E}^{\mathcal{F}}$	${E}^{\mathcal{A}\mathcal{B}\mathcal{C}}$	${E}^{\mathcal{G}\mathcal{A}}$	${E}^{\mathcal{A}\mathcal{G}}$
$4$	$370$	$24.586$	$34.797$	$35.206$
$5$	$472$	$30.890$	$43.737$	$44.268$
$6$	$574$	$37.193$	$52.676$	$53.329$
$7$	$676$	$43.395$	$61.616$	$62.391$
$8$	$778$	$49.802$	$70.555$	$71.453$
$9$	$880$	$56.103$	$79.494$	$80.575$
$10$	$982$	$62.406$	$88.434$	$89.515$
$11$	$1084$	$68.710$	$97.373$	$98.639$
$12$	$1186$	$75.013$	$106.313$	$107.701$
$13$	$1288$	$81.316$	$115.252$	$116.762$

**Table 4:** Expected values of indices for ${m}_{1}=\frac{1}{4}$.

$z$	${E}^{\mathcal{F}}$	${E}^{\mathcal{A}\mathcal{B}\mathcal{C}}$	${E}^{\mathcal{G}\mathcal{A}}$	${E}^{\mathcal{A}\mathcal{G}}$
$4$	$370$	$24.6071$	$34.777$	$35.226$
$5$	$472$	$30.920$	$43.707$	$44.299$
$6$	$574$	$37.234$	$52.636$	$53.371$
$7$	$676$	$43.547$	$61.565$	$62.443$
$8$	$778$	$49.860$	$70.494$	$71.515$
$9$	$880$	$56.174$	$79.424$	$80.587$
$10$	$982$	$62.487$	$88.353$	$89.659$
$11$	$1084$	$68.801$	$97.282$	$98.732$
$12$	$1186$	$75.114$	$106.212$	$107.804$
$13$	$1288$	$81.427$	$115.141$	$116.876$

### Theorem 5

*Let* $${z}\ge 2,$$* then*

$$E[\mathcal{F}(\mathcal{R}\mathcal{C}\mathcal{O}\mathcal{C}\left(z;{m}_{1},{m}_{2},{m}_{3})\right)]>E[\mathcal{A}\mathcal{G}(\mathcal{R}\mathcal{C}\mathcal{O}\mathcal{C}\left(z;{m}_{1},{m}_{2},{m}_{3})\right)].$$

### Proof

 It is true for ${z}=2$. Now, let ${z}>2$, by using Theorem 1 and 2, we have

$$E[\mathcal{F}\left(\mathcal{R}\mathcal{C}\mathcal{O}\mathcal{C}\left(z;{m}_{1},{m}_{2},{m}_{3})\right)\right]-E[\mathcal{A}\mathcal{G}(\mathcal{R}\mathcal{C}\mathcal{O}\mathcal{C}\left(z;{m}_{1},{m}_{2},{m}_{3})\right)]$$

$$=z\left[102\right]-38-\left[z\left[\left(\frac{12-5\sqrt{6}}{6}\right){m}_{1}+\frac{15+5\sqrt{6}}{3}\right]-\left(\frac{12-5\sqrt{6}}{3}\right){m}_{1}+\frac{9-5\sqrt{6}}{3}\right]$$

$$=\left({z}-2\right)\left[\frac{321+5\sqrt{6}}{3}+\left(\frac{12-5\sqrt{6}}{6}\right){m}_{1}\right]+179+5\sqrt{6}$$

$$=\frac{\left({z}-2\right)}{3}\left[321+5\sqrt{6}+\left(\frac{12-5\sqrt{6}}{2}\right){m}_{1}\right]+179+5\sqrt{6}$$

$$>0$$

$$\because {z}\ge 2\,\, and\,\, 0\le {m}_{1}\le 1.$$

### Theorem 6

*If* $${z}\ge 2,$$* then*

$$E[\mathcal{A}\mathcal{G}(\mathcal{R}\mathcal{C}\mathcal{O}\mathcal{C}\left(z;{m}_{1},{m}_{2},{m}_{3})\right)]>E[\mathcal{G}\mathcal{A}(\mathcal{R}\mathcal{C}\mathcal{O}\mathcal{C}\left(z;{m}_{1},{m}_{2},{m}_{3})\right)].$$

### Proof

 It is true for $\mathcalligra{z}=2$. Let $\mathcalligra{z}>2$, by using Theorem 2 and 3, we have.

$$E[\mathcal{A}\mathcal{G}\left(\mathcal{R}\mathcal{C}\mathcal{O}\mathcal{C}\left(z;{m}_{1},{m}_{2},{m}_{3})\right)\right]-E[\mathcal{G}\mathcal{A}(\mathcal{R}\mathcal{C}\mathcal{O}\mathcal{C}\left(z;{m}_{1},{m}_{2},{m}_{3})\right)]$$

$$=\left[z\left[\left(\frac{12-5\sqrt{6}}{6}\right){m}_{1}+\frac{15+5\sqrt{6}}{3}\right]-\left(\frac{12-5\sqrt{6}}{3}\right){m}_{1}+\frac{9-5\sqrt{6}}{3}\right]-\left[z\left[\left(\frac{10-4\sqrt{6}}{5}\right){m}_{1}+\frac{25+8\sqrt{6}}{5}\right]-\left(\frac{20-8\sqrt{6}}{5}\right){m}_{1}+\frac{15-8\sqrt{6}}{5}\right]$$

$$=\left({z}-2\right)\left[\frac{\sqrt{6}}{15}+\left(\frac{\sqrt{6}}{30}\right){m}_{1}\right]+\frac{\sqrt{6}}{15}$$

$$=\frac{\left({z}-2\right)}{15}\left[\sqrt{6}+\left(\frac{\sqrt{6}}{2}\right){m}_{1}\right]+\frac{\sqrt{6}}{15}$$

$$>0$$

$$\because {z}\ge 2\,\, and\,\, 0\le {m}_{1}\le 1.$$

### Theorem 7*.*

$$E[F\left(\mathcal{R}\mathcal{C}\mathcal{O}\mathcal{C}\left(z;{m}_{1},{m}_{2},{m}_{3})\right)\right]>E[\mathcal{A}\mathcal{G}(\mathcal{R}\mathcal{C}\mathcal{O}\mathcal{C}\left(z;{m}_{1},{m}_{2},{m}_{3})\right)]>E[\mathcal{G}\mathcal{A}(\mathcal{R}\mathcal{C}\mathcal{O}\mathcal{C}\left(z;{m}_{1},{m}_{2},{m}_{3})\right)]>E[\mathcal{A}\mathcal{B}\mathcal{C}(\mathcal{R}\mathcal{C}\mathcal{O}\mathcal{C}\left(z;{m}_{1},{m}_{2},{m}_{3})\right)]$$.

## Data Availability

All data generated or analysed during this study are included in this published article [and its supplementary information files].
